# Considerations for Measurements of Aggregate PFAS Exposure in Precision Environmental Health

**DOI:** 10.1021/acsmeasuresciau.4c00052

**Published:** 2024-10-22

**Authors:** Katherine E. Manz

**Affiliations:** † Department of Environmental Health Sciences, University of Michigan, Ann Arbor, Michigan 48109, United States

**Keywords:** Per- and polyfluoroalkyl substances, Aggregate exposure, Precision environmental health, Total fluorine, Total organic fluorine

## Abstract

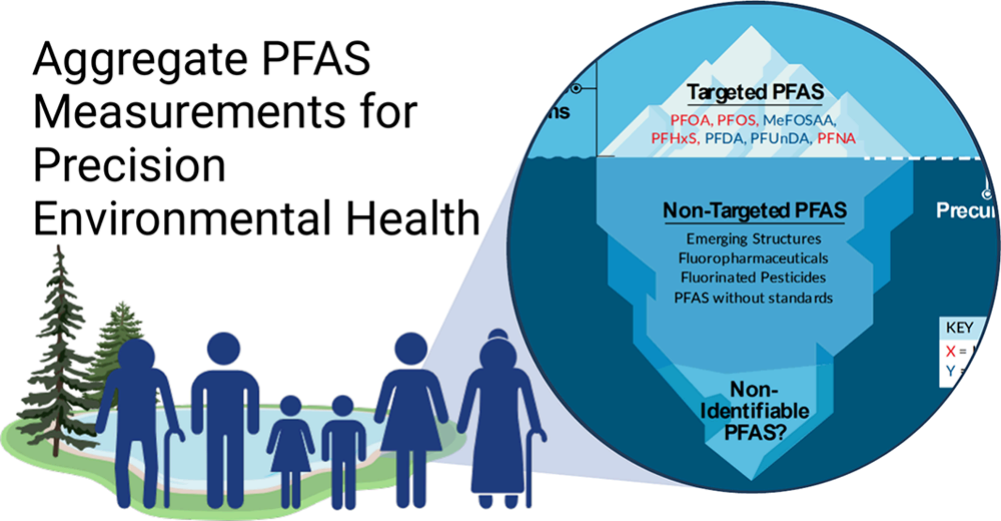

Per- and polyfluoroalkyl substances (PFAS) have become a major focus of research due to their widespread environmental presence and adverse health effects associated with human exposure. PFAS include legacy and emerging structures and are characterized by a range of functional groups and carbon–fluorine chains that vary in length (from fewer than 3 carbons to more than 7 carbons). Research has linked PFAS exposure to an array of health concerns, ranging from developmental and reproductive disorders to immune system impairments and an increased risk of certain cancers. In this new era of personalized health, measuring markers of PFAS exposure in human biospecimens is an important part of environmental public health surveillance. PFAS are typically measured in human blood and tissues using targeted approaches, which quantify individual PFAS structures using specific instrumentation. The diversity and complexity of PFAS, the limitations of the targeted approaches due to the sheer number of structures, and the absence of publicly available analytical standards pose significant challenges for measurement methodologies. This perspective aims to describe aggregate PFAS exposure measurements and their potential for use in precision medicine applications including a discussion of the limitations and potential benefits of these aggregate measurements. As public health organizations, healthcare professionals, and the public look for guidance regarding the safe use of and exposure to PFAS, in a pragmatic cost-effective manner, the dynamic field of measurement science is poised to respond with innovative technological solutions to an important public health need.

## 1 Introduction

**Figure 1:**
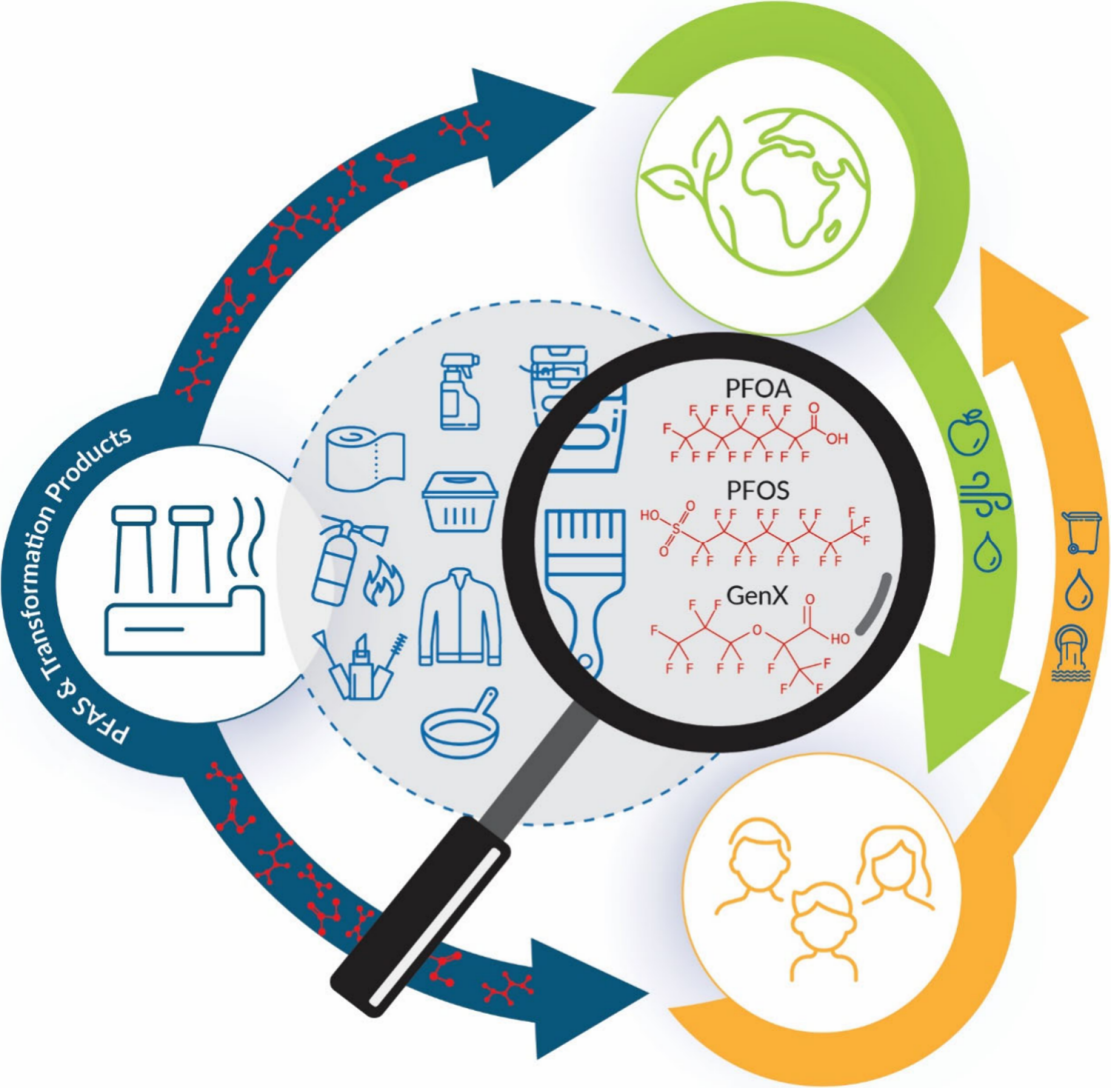
Schematic showing the cycle of PFAS. PFAS are synthesized for incorporation into commercially available products. Contaminated soils, groundwater, and waste from industry production and use and human use of PFAS-containing products impact human and environmental health. Humans release PFAS into the environment through waste and wastewater, where the chemicals accumulate. In turn, the accumulation of PFAS from industry and waste causes exposure through contaminated sites (including sites contaminated with Aqueous Film-Forming Foam (AFFF)), air pollution, water pollution, and contaminated food.

Per- and polyfluoroalkyl substances (PFAS), due to their pervasive presence in our environment and adverse health effects, have emerged as a critical focus of research and concern for human health.^1−4^ These chemicals, characterized by their strong carbon–fluorine bonds, have been extensively utilized in various industrial and consumer products for decades, contributing to their ubiquitous presence in the environment.^5,6^ Due to their heat, oil, and water resistant properties, PFAS are used in nonstick cookware (e.g., Teflon), water-repellent fabrics, food packaging (e.g., fast food wrappers and popcorn bags), aqueous film-forming foams (AFFF) for firefighting, cosmetics, medical devices, cleaning products, paints and stains, semiconductors, and more (Figure 1).

The PFAS chemical class has been estimated to contain thousands to millions of structures—the EPA’s CompTox Chemicals Dashboard currently contains 14,735 PFAS in the PFAS Structure Lists, while PubChem contains over 7 million PFAS according to the Organisation for Economic Co-operation and Development (OECD) PFAS definition (any chemical that contains at least one CF_2_ or CF_3_ moiety).^7−11^ However, the number of commercially available analytical standards is substantially reduced due to patent infringement lawsuits preventing companies from producing them.^12^ “Legacy” PFAS refer to the first generation of these chemicals that were produced extensively since the 1950s, most prominently perfluorooctanoic acid (PFOA) and perfluorooctanesulfonate (PFOS). Replacement of legacy PFAS began in the early 2000s when they were voluntarily withdrawn or phased out of production in the United States and Europe due to the environmental persistence and health effects. Globally, serum concentrations of the long-chain, legacy chemicals PFOA and PFOS decreased after the voluntary phase-out.^13−17^ “Emerging PFAS” are newer-generation PFAS that are gaining attention due to their increased detection in the environment and potential health impacts. In some cases, emerging PFAS replace the use of legacy PFAS. PFAS are distinguished by the functional groups (i.e., carboxylic acids, sulfonic acids, and ether structures) and straight or branched carbon–fluorine chains classified as either ultrashort (≤3 carbons), short (4–6 carbons), or long (≥7 carbons). In light of the limited toxicity information available for emerging structures, the dynamic changes in usage (structure, quantity, application, geography) results in poorly understood exposure.^18−24^ Concerningly, emerging PFAS are used in commercial products (e.g., 6:2 fluorotelomer phosphate diester in toilet paper,^25^ fluorotelomer methacrylates in cosmetics,^26^ and shorter chain PFAS in clothes^27^) without careful consideration or discussion of the potential environmental and human toxicological impacts.^28^ Thus, human exposure to PFAS may be underestimated, posing a potential risk to human health.

Research has linked PFAS exposure to an array of health concerns, ranging from developmental and reproductive disorders to immune system impairments and increased risks for certain cancers. PFAS are considered a public health concern by several regulatory agencies, including the Centers for Disease Control (CDC) and Prevention’s National Center for Environmental Health, the Agency for Toxic Substances and Disease Registry (ATSDR), and Environmental Protection Agency (EPA).^29−31^ In early life, epidemiological studies have linked prenatal PFAS exposure to a range of adverse health outcomes, including gestational weight gain, low birth weight, preterm birth, reduced vaccine response, and metabolic alterations.^32−37^ PFAS have also been linked to cancers, including kidney and testicular, thyroid disease, increased cholesterol levels, and liver damage.^38−44^ Although much research has focused on legacy PFAS, especially PFOA and PFOS, we are still learning about the health effects of emerging PFAS, as the distribution of these chemicals in the human body is structure-specific.^45^ However, recent data suggests that emerging PFAS are associated with some of the same adverse health effects originally described for legacy PFAS.^46−48^ As our understanding of the health effects of PFAS on humans increases, so does the urgency to regulate and monitor human exposure to them.

Precision environmental health is an emerging field focusing on how genetic, environmental, and lifestyle factors influence health outcomes across populations, time, and life stages.^49^ This approach emphasizes the individual patient’s uniqueness and desire for personalized interventions that prevent the adverse health effects of exposures, including PFAS exposure. The rise of precision medicine has the potential to revolutionize healthcare through approaches such as personalized chemical exposure assessments that can be integrated into medical records. In this new era of personalized health, measuring markers of PFAS exposure in human specimens is an important part of environmental public health surveillance. PFAS are typically measured in human blood and tissues using targeted approaches, which quantify individual PFAS structures using specific instrumentation (liquid chromatography paired with mass spectrometry (LC-MS)). Targeted methods for measuring PFAS concentrations are standardized (e.g., EPA Method 1633,^50^ CDC Method 6304.09^51^) and accessible. However, they are limited to PFAS with commercially available analytical standards. Thus, targeted methods tend to quantify legacy PFAS. A major challenge to accurately assessing exposure to emerging PFAS is that they are not routinely quantified in traditional targeted approaches, often due to the lack of availability of analytical standards. There is a growing recognition of the need to comprehensively understand an individual’s total PFAS exposure—beyond singular assessments of specific structures.

The diversity and complexity of PFAS, with numerous compounds of varying structures and unique functional groups, pose a substantial challenge for measurement methodologies. Further complicating the challenge is that emerging and many legacy PFAS are commonly proprietary chemicals, and patent protection limits the manufacturers’ ability to produce analytical standards, which impedes scientists’ ability to measure them, confirm their prevalence, and understand their health effects. Accurate and comprehensive measurement techniques to assess aggregate PFAS exposure are pivotal for understanding the prevalence, distribution, and potential risks associated with legacy and emerging PFAS. A holistic, aggregate exposure approach acknowledges the dynamic nature of PFAS exposure and considers the cumulative impact of multiple chemicals and their potential synergistic effects on human health. This perspective intends to provide an overview and assessment of the current methodology in the context of dynamic PFAS use scenarios, increased exposure, and growing concern for the public health impact of these chemicals.

## 2 Aggregate PFAS Measurements

**Figure 2:**
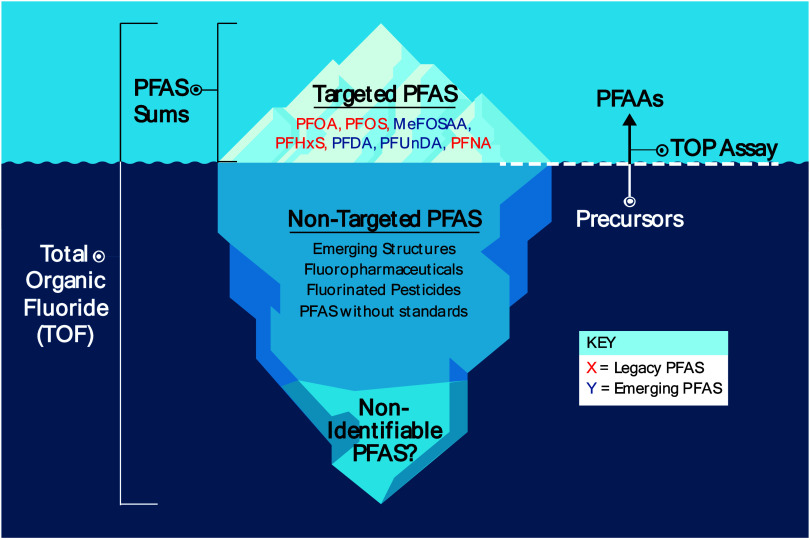
PFAS iceberg. Above the surface lies targeted PFAS that are widely recognized and include both legacy and emerging structures. Below the surface are less studied PFAS including those discoverable by Non-Targeted Analysis. Aggregate measures for PFAS exposure assessment cover different parts of the PFAS iceberg.

Several measurements have emerged recently to estimate total PFAS exposure and have potential use in precision medicine applications. These methods include PFAS Sums, Non-Targeted Analysis (NTA), the Total Oxidizable Precursor (TOP) assay, and Total Fluorine (TF) and Total Organic Fluorine (TOF) measurements (Table 1). The methods measure parts of the “PFAS iceberg” (Figure 2), which depict how legacy PFAS and other known PFAS constitute only a small fraction of the broader problem. The tip of the iceberg represents PFAS that are widely recognized and studied, including PFOA and PFOS. These PFAS have been the focus of regulatory action and public awareness. Beneath the water’s surface lies the vast majority of PFAS that are less understood, including emerging structures, fluoropharmaceuticals, fluorinated pesticides, PFAS without analytical standards, and PFAS that we may not be able to identify. Addressing the entire spectrum of PFAS for precision environmental health requires comprehensive strategies that go beyond the currently regulated substances, including those represented in Figure 2.

**Table 1:** Measurements Used for Aggregate PFAS Exposure Assessment

Aggregate PFAS Measurement	Description
PFAS Sums (Targeted Analysis)	Relies on traditional targeted LC-MS analysis to first determine PFAS concentration in serum. Then, the concentrations of individual PFAS are summed.
Non-Targeted Analysis (NTA)	Relies on high-resolution mass spectrometry (HRMS) to screen for legacy and emerging PFAS that may or may not have analytical standards available.
Total Oxidizable Precursor (TOP) Assay	An assay that oxidizes precursor PFAS into perfluoroalkyl acids (PFAAs) and quantifies total concentration in the oxidized sample using targeted LC-MS analysis of PFAAs.
Total Fluorine (TF)	Measurements that aim to measure all fluorine in a sample, including inorganic, nonextractable fluorine, and organically bound fluorine. Particle-Induced Gamma Ray Emission (PIGE) measures TF.
Total Organic Fluorine (TOF)	Measurements that aim to measure all organically bound fluorine within a sample. Relies on Combustion Ion Chromatography (CIC) to measure extractable organic fluorine (EOF) and Adsorbable organic fluorine (AOF).

### PFAS Sums

Currently, summing the concentrations of PFAS detected in targeted LC-MS analytical methods is the most common practice in public health research for aggregate exposure. PFAS Sums provide a comprehensive, practical, and communicable measure of the total burden from the specific PFAS measured. One example is the approach from Recommendation 5-3 in the National Academies of Sciences, Engineering, and Medicine (NASEM) report “Guidance on PFAS Exposure, Testing, and Clinical Follow-Up (2022)” that sums the seven PFAS currently measured in the National Health and Nutrition Examination Survey (NHANES).^52^ The PFAS considered in this approach are PFOA, PFOS, Methylperfluorooctane sulfonamidoacetic acid (MeFOSAA), Perfluorohexanesulfonic acid (PFHxS), Perfluorodecanoic acid (PFDA), Perfluoroundecanoic acid (PFUnDA), and Perfluorononanoic acid (PFNA). According to these guidelines, serum or plasma sum concentrations should inform clinical care as follows: < 2 ng/mL (not expected to have adverse health effects), 2–20 ng/mL (potential for adverse health effects, especially for vulnerable populations), and >20 ng/mL (increased risk of health effects). Ultimately, PFAS Sums are limited to PFAS that can be quantified in targeted analysis. Thus, when PFAS Sums are used in epidemiological studies or for personal monitoring, they may encompass different ranges of PFAS structures depending on the targeted methodology used by the analytical laboratory.

An important limitation of PFAS Sums in environmental precision health studies is that the biological relevance of the concentration, toxicity, and health effects of individual PFAS may differ greatly. For instance, the impact of a particular PFAS could be masked by the presence of another PFAS that is more abundant but has lower toxicity. Therefore, nuanced approaches to evaluate exposure and risk, such as potency-weighted PFAS sums, could be considered. Toxic equivalencies have been previously applied to creating aggregate exposure summaries to dioxins and dioxin-like chemicals.^53^ The total toxic equivalency of a mixture (TEQ) is calculated by weighing the concentrations of chemicals by their relative toxicities. For PFAS, this may provide a more accurate representation of the potential health risks posed by the mixture of PFAS. However, considerable research is required to establish potency factors for the numerous PFAS structures that exist and to determine how additive and nonadditive interactions could be considered in the weighting. Incorporating potency-weighted sums into PFAS epidemiological studies would ultimately enhance our understanding of the health impacts of these pervasive environmental contaminants.

### Non-Targeted Analysis

Non-Targeted analysis (NTA) is an analytical approach that aims to capture as many chemical species as possible and relies on high-resolution mass spectrometry (HRMS).^54^ NTA has become pivotal for identifying PFAS in the environment; for example, perfluoro-2-propoxypropanoic acid (also known as hexafluoropropylene oxide dimer acid, HFPO–DA, and the trade name “GenX”) was discovered in the Cape Fear River using NTA^55^ and is now included in the EPA’s National Primary Drinking Water Regulation (NPDWR) standards announced in April 2024. In human blood, NTA has discovered both legacy and emerging structures, including perfluoroalkyl acids (PFOA and PFOS), perfluoropolyether carboxylic acids (PFECA), carboxylic acid-perfluoroalkyl sulfonamides (CA-PFSMs), and fluorotelomer sulfonic acids (FTS).^56,57^ Unlike targeted PFAS methods (EPA Method 1633, for example), NTA is not yet standardized. However, PFAS NTA can be implemented into the targeted PFAS workflows that use HRMS and collect MS^2^ data.^58^ For example, using a targeted HRMS workflow with proper QA/QC and blanks could save time and resources and provide enhanced identification of emerging PFAS. Recently, PFAS NTA has been performed using gas chromatography (GC) HRMS^59^ to discover emerging, volatile PFAS.

For LC-HRMS NTA, liquid chromatography and mass spectral heuristics have enabled PFAS discovery. Using the accurate mass collected by HRMS, we use the Kendrick mass defect (KMD), which is the difference between the exact mass and the nominal mass of a detected feature, can help identify fluorine-containing compounds.^60^ When homologous series of PFAS with the same functional group and varying chain length are present within a sample, the liquid chromatography retention time of each PFAS is ordered sequentially, with short-chain PFAS eluting early and long-chain PFAS eluting later in an analytical run (if the mobile phase starts with the more polar solvent), and the mass-to-charge (*m*/*z*) ratio of the homologs varies by 49.9968 (CF_2_). Recent PFAS-specific NTA software, including FluoroMatch and FindPFΔ*S*, have incorporated these heuristics into their annotation.^61−63^ It is also essential that NTA workflows incorporate libraries of MS^2^ spectra built from reference standards for higher confidence annotations.^60^ With this evidence (KMD, a homologous series in retention time order, MS^2^ spectra), a reasonable chemical formula or structure can be proposed, but it can still be difficult to confirm the structure without analytical standards.

NTA has helped scientists overcome the limitations of targeted analysis, transforming our approach in precision environmental health and increasing the ability to identify new potentially harmful exposures. The application of NTA in precision medicine for aggregate PFAS exposure assessment still requires standardization for individualized healthcare scenarios and may always have limitations. NTA provides peak areas of the *m*/*z* detected, which vary from instrument to instrument, and a mass spectrum with tentative identification. Importantly, these peak areas are unitless and more difficult to interpret than the concentrations obtained by targeted analysis. For NTA to be fully quantitative, a complete library of analytical standards for all possible structures would need to be available, and this likely will not be achieved. Thus, normalization of these intensities through semiquantitative analysis (using the calibration curve collected for a PFAS with a similar structure and analytical standard) or statistical standardization can provide actionable data for precision health applications. Further, a pivotal effort in harmonizing NTA across laboratories is the standardization of references through concurrently analyzed pooled reference samples so that laboratories can ensure a higher degree of consistency and comparability in their data. This practice not only mitigates interlaboratory variability but also enhances the reliability and reproducibility of NTA results. The potential benefits of improved standardization and data harmonization might pave the way for broader applications in the future. While NTA requires extensive labor and expertise, integrating NTA into precision environmental health research would allow us to have a more comprehensive understanding of the PFAS exposome, which changes as new chemicals are brought into production. Although NTA cannot provide concentration values in the absence of an analytical standard, NTA can reveal the presence of potentially harmful chemical substances and drive the trajectory toward precision environmental health.

### Total Oxidizable Precursor Assay

The Total Oxidizable Precursor (TOP) assay is designed to estimate the total concentration of PFAS in a sample, including measurable PFAS and their precursors. Precursors are compounds that can transform into PFAS through environmental or biological processes. The TOP assay relies on a hydroxyl radical-based oxidation reaction to transform precursor PFAS into reaction end products (perfluoroalkyl acids (PFAAs)).^64^ In the assay, a sample is pH adjusted to an alkaline pH, digested using heat-activated persulfate to convert precursors to perfluoroalkyl acids (PFAAs), extracted using solid-phase extraction, and analyzed using targeted LC-MS to quantify the sum of perfluoroalkyl carboxylic acids (PFCAs) and perfluoroalkyl sulfonic acids (PFSAs). The increase in the PFAA concentration after oxidation indicates the presence of precursor PFAS, some of which could be metabolized to legacy PFAS and others to emerging PFAS; therefore, the TOP assay provides a quantitative estimate of oxidizable precursors in a sample when paired with targeted analysis before and after the assay. The TOP assay was originally developed for large volumes of water but has been applied to human serum samples.^65^ In human serum, the TOP assay has indicated whether or not a human was exposed to unknown oxidizable precursors, enhancing holistic assessment of human exposure to PFAS.^65^ For the application of the TOP assay in precision environmental health, the method still requires optimization and careful consideration. Human samples are a complex biological matrix containing proteins, lipids, metabolites, and other biomolecules. The matrix may interfere or quench the oxidation reaction, causing incomplete oxidation of the PFAS precursors or suppression of PFAS in LC-MS analysis. However, the assay is straightforward and accessible to laboratories already performing targeted analysis with LC-MS. With further development, the TOP assay could be used to assess human exposure to PFAS and PFAS precursors, providing a more comprehensive understanding of the total PFAS burden in the body.

### Total Fluorine

**Figure 3:**
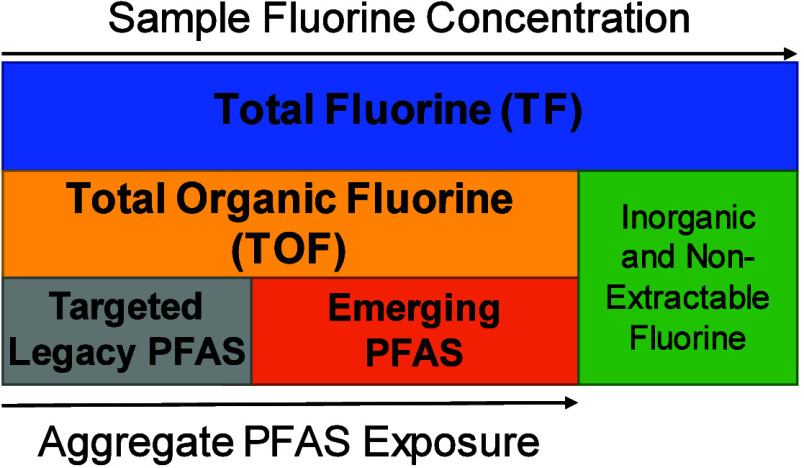
Schematic representing the constituents of total fluorine in a sample.

Total Fluorine (TF) can be highly valuable in the field of precision environmental health. TF encompasses organic fluorine, including legacy and emerging PFAS, and inorganic fluorine and nonextractable fluorine (Figure 3). TF can be measured using Particle-Induced Gamma Ray Emission (PIGE). PIGE measures the concentration of total fluorine in a sample using gamma-ray emissions but cannot differentiate organic and inorganic fluoride. One benefit to PIGE is that the technique is nondestructive, allowing the sample to be used for further analysis if needed. PIGE has been applied to measure TF consumer products;^26,66^ however, examples of applications to human biospecimens are limited. While PIGE is highly sensitive for detecting fluorine at low concentrations, there are limitations and considerations for its use in precision environmental health and epidemiology for aggregate PFAS exposure assessment. Human biospecimens contain high concentrations of inorganic fluorine; therefore, extraction methods prior to PIGE (such as solid-phase extraction) are required to isolate PFAS in the sample from inorganic fluorine. Further research is needed to refine these extraction methods and validate their effectiveness across biospecimens to understand their utility in precision environmental health applications. It is very important that future research on PIGE analysis of biospecimens is paired with other organic fluorine and/or targeted PFAS measurements to consider the contributions of inorganic fluorine to these measurements, even after extraction.

### Total Organic Fluorine

Total Organic Fluorine (TOF) measurements are useful for estimating aggregate PFAS exposure, and when paired with targeted analysis, provide a quantitative assessment of the relative importance of different PFAS exposures.^3^ TOF is determined by measuring the total fluorine in a sample and subtracting the measured inorganic fluorine in a sample (Figure 3). TOF encompasses legacy PFAS (i.e., PFOA and PFOS) and emerging PFAS (Figure 3), but does not differentiate them; thus, TOF is related to total PFAS exposure and can be used as an aggregate measure to the PFAS mixture.^47,67^ TOF is commonly measured using Combustion Ion Chromatography (CIC). For CIC measurements, TOF can be extracted from a sample (“Extractable Organic Fluoride”) (EOF)) or absorbed onto carbon from a sample (“Adsorbable Organic Fluorine” (AOF)). Thus, it is important to acknowledge that EOF and AOF will have biases as to what is extractable or adsorbable, respectively. CIC uses combustion at ∼1000 °C to convert the organic fluorine to inorganic fluoride, which is then measured by a conductivity detector. For EOF, the liquid extract is injected and combusted by the CIC; while for AOF, the carbon material that the PFAS was adsorbed onto is combusted. While the application of AOF for PFAS exposure assessment in human biospecimens is limited, EOF has been applied for assessing aggregate PFAS exposure in several studies.^67−69^ For precision environmental applications, epidemiological studies have yet to link TOF to health outcomes, and more research is needed in this area to determine if TOF measurements are applicable to precision environmental health assessments. Importantly, the TOF does not differentiate between individual PFAS, which complicates the inclusion of toxicity weights. Similar to PIGE, future research studies using TOF measurements in epidemiological studies should continue to pair this method with targeted and non-targeteda pPFAS measurements to better understand the composition of TOF.

## 3 Limitations of Aggregate PFAS Measurements

The primary goal of aggregate PFAS measurements is to better understand total PFAS exposure, so that the risks associated with exposure can be better estimated and interpreted for intervention in precision health. For this purpose, results in human serum can be considered by clinicians and medical practitioners. However, aggregate PFAS measurements do not come without limitations. The first limitation is differentiating fluorine species in methods that are not traditional targeted LC-MS analysis (e.g., PFAS Sums), as these methods cannot identify or quantify new or unknown PFAS compounds. Differentiation of fluorine may be important when non-PFAS, organically bound fluorine, is highly concentrated in a sample. For example, while organically bound fluoride chemicals are not present in nature, anthropogenic fluorinated pharmaceuticals and pesticides may contribute fluorine to TOF. Previous studies have estimated that 18–25% of all pharmaceuticals approved since 1991 contain at least one fluorine atom; while 1.1–30% of fluoropharmaceuticals are PFAS.^70−72^ However, pharmaceuticals are not expected to have long half-lives in the human body (in comparison to the half-lives of PFAS); therefore, understanding a patient’s pharmaceutical use could be useful in determining whether or not a TOF is suitable for assessing aggregate exposure, and the contributions of pharmaceuticals to TOF should be considered in the design of large epidemiological studies. Similarly, biospecies also contain inorganic fluorine. Therefore, it is important to ensure that inorganic fluoride does not cause any interference in the measurement or has been removed by extraction prior to measurement. For example, if a sample is measured by EOF, the sample cleanup using solid phase extraction could remove residual inorganic fluoride or the fluoride content of the extract could be measured with and without combustion (with precombustion fluoride approximating the inorganic contribution). Pairing NTA with the TOF measurement could be very beneficial for understanding the contribution and prevalence of non-PFAS to aggregate exposure. Although NTA has limitations in determining exact molecular structures without a chemical standard, a chemical formula may provide sufficient information for determining whether or not a chemical is a PFAS, especially when applying the definition of PFAS that considers 30% of the molecules within a formula must be fluorine.^73^

The second limitation of using aggregate PFAS measurements is that the sample volume available or the limits of detection of each measurement could limit the feasibility of each measurement. Recently, targeted LC-MS methods have been developed to use as little as 30 μL of sample with detection limits of less than 0.5 ng/mL for individual PFAS.^74^ CDC method 6304.09 uses 50 μL of serum with detection limits of 0.1 ng/mL.^51^ The limits of detection for TOF measurements are not well-established for serum, and research is needed to establish these and the minimum amount of serum that can be used for the measurement. Compared with targeted analysis, TOF measurements typically exhibit higher limits of detection than targeted LC-MS approaches, therefore requiring higher sample volumes to achieve limits of detection adequate for biomonitoring. In addition to requiring greater sample volumes, the TOF faces significant challenges with background contamination (including residual inorganic fluoride) and matrix effects, which can interfere with the accuracy and reliability of the readings. Background levels in TOF measurements can obscure signals from low-abundance PFAS, making it difficult to achieve the precision needed for effective biomonitoring. This complicates the detection and quantification process, especially in complex biological matrices, such as serum. These limitations underscore the need for further research and methodological optimization to enhance the sensitivity and accuracy of TOF measurements for PFAS analysis in biomonitoring studies.

Finally, the third limitation is that too few epidemiological studies linking exposure to health outcomes include aggregate PFAS measurements. While PFOA and PFOS have been associated with several adverse health outcomes, the potential toxicity of the other members of the PFAS chemical class remains uncertain. Replacement of legacy PFAS is a growing environmental health issue. In serum from Swedish women collected between 1996 and 2012, the increased EOF exposure was observed, but the contribution by targeted PFAS (61 PFAS were targeted, including PFOA and PFOS) declined by 3.5% per year.^75^ This increase in EOF and decline in targeted PFAS suggest that exposure to emerging PFAS is increasing. As the number of PFAS structures continues to grow, it will be very challenging to continue to measure and link individual PFAS levels to health outcomes. The process of assessing PFAS toxicity at the level of individual structures (targeted) will only slow our ability to regulate these harmful chemicals.

## 4 Conclusions

Many industries use PFAS in their products and processes. However, PFAS use and disposal are not yet regulated or transparent to consumers. While PFAS regulations are essential for preventing potential health and other environmental risks, the scientific community must offer biomonitoring of PFAS in a way that keeps up with the ongoing replacement of legacy PFAS. Aggregate methods are our best hope for understanding total exposure in precision environmental health applications, which aims to provide highly detailed and personalized information regarding an individual’s exposure—including PFAS. The aggregate measures discussed in this perspective hold promise for understanding exposures in the shorter term and, when adapted iteratively as research in the field progresses, can help preserve accessibility to exposure information over time. In addition to providing a total exposure assessment and allowing us to assess cumulative risk more effectively, aggregate PFAS measurements streamline exposure assessment, making it more practical and potentially cost-effective to monitor overall PFAS levels. In the health risk assessment, aggregate measurements help to understand the cumulative health risks associated with PFAS exposure. This is crucial because the combined effect of multiple PFAS compounds can be more harmful than that of individual compounds alone. Despite the loss of specificity with total organic fluorine measurements, the total organic fluorine concentration is a more straightforward way to report PFAS to exposed individuals, which may help promote effective public health responses.

As individuals seek more specific information about their PFAS exposure in the future, the limitations of aggregate PFAS measurements will become more apparent. People will likely demand more precise data to understand their specific exposure to different PFAS compounds and their potential health risks. Optimizing TOF methods and integrating them with targeted analysis could bridge this gap, enabling more detailed and accurate biomonitoring results. Such advancements will be crucial for delivering tailored health recommendations and interventions based on precise environmental exposure data, advancing the field of precision environmental health.
